# Study on the mechanism of TP53 degradation by SFN ubiquitination affecting locally advanced TC progression

**DOI:** 10.3389/fchem.2026.1816666

**Published:** 2026-06-29

**Authors:** Ying Peng, Bin Liu, Ting-Ting Yang, You-Wen Su, Lin Zhang, Zhi-Zhong Dong, Chang Diao, Yan-Jun Su, Ruo-Chuan Cheng

**Affiliations:** 1 Thyroid Disease Diagnosis and Treatment Center, First Affiliated Hospital of Kunming Medical University, Kunming, Yunnan, China; 2 Kunming Medical University, Kunming, Yunnan, China

**Keywords:** advanced thyroid cancer, SFN, TP53, tumor metastasis, ubiquitination

## Abstract

**Aim:**

Revealing the molecular mechanism of metastasis of advanced thyroid cancer and transforming it into neoadjuvant targeted therapy, to get a great potential to reduce operative mortality and improve clinical prognosis in locally advanced Thyroid Cancer (TC) patients.

**Methods:**

Serological tests were conducted to detect the expression level of natural TF-Ab and the sialacidification level of Thomson friedenreich antibody (TF-Ab) in the population; Analysis of downstream acting proteins after exposure to TF-Ag by IP binding mass spectrometry. Clinical histological immunohistochemistry confirmed the expression of the target protein in locally advanced TC tissues. *In vitro* and *in vivo* experiments verified the influence of target protein expression regulation on the biological functions of TC cells and tumor progression.

**Results:**

The expression of natural TF-Ab in tumor patients is lower than that in healthy people, while there was no significant difference in the level of sialacidification among antibodies; IP combined with mass spectrometry showed that SFN was a stable and highly expressed molecular interaction protein after exposure to TF-Ag. Clinical histology verified that SFN was significantly higher expressed in locally advanced TC tissues than in inert TC tissues (T1 stage). Cell biology experiments have demonstrated that SFN expression regulation can significantly affect the biological function of TC cells and change the differentiation ratio of CSCs. Intervention of SFN expression *in vivo* tumor formation experiments in nude mice can significantly inhibit tumor progression.

**Conclusion:**

The effect of TP53 degradation by SFN ubiquitination on the differentiation of CSCs is a potential molecular mechanism for the widespread metastasis of locally advanced TC.

## Introduction

Thyroid Cancer (TC) is the most common endocrine malignancy. Surgery, chemotherapy, radiotherapy, and radioactive iodine (RAI) therapy are standard TC treatments (Zhang et al., 2023). However, statistical analysis found that the 5-year relative survival rates of TC patients with local non-spread, regional metastasis and distant metastasis were 99.9%, 98.3% and 55.3%, respectively (Thyroid Cancer, 2023), indicating that tumor metastasis is still a major challenge for TC treatment. At present, many scholars refer to this aggressive TC accompanied by extensive metastasis and difficult radical resection as advanced TC (Shonka et al., 2022). Advanced TC can obviously invade the surrounding important structures, and has a poor prognosis (Jannin et al., 2025). With a wide range of operations, involving the resection and reconstruction of vital organs, surgical trauma and risk are relatively large, and the incidence of complications is relatively high, which is the fundamental cause of death affecting the prognosis of TC patients (Agosto et al., 2023). In recent years, molecular detection of advanced and/or metastatic cancers has become an essential component of modern precision cancer therapy (Brose et al., 2024; Pitoia et al., 2024; Wells et al., 2012). Metastasis is a key factor involved in the progression and prognosis of advanced thyroid cancer (Komatsuda et al., 2023). Therefore, revealing the molecular mechanism of metastasis of advanced thyroid cancer and transforming it into neoadjuvant targeted therapy has great potential to reduce operative mortality and improve clinical prognosis in locally advanced TC patients. However, at present, there is no effective superior treatment plan for locally advanced TC patients in clinical diagnosis and treatment, and it is urgent to explore more efficient, sensitive and specific treatment means.

Based on previous research findings, which are consistent with results from other tumor studies, the tumor-associated Thomsen-Friedenreich antigen (TF-Ag) appears to be closely associated with the metastatic mechanisms of TC. Antibodies targeting the TF-Ag have demonstrated efficacy in inhibiting TC progression (Peng et al., 2020). However, the clinical translation of antibody-based tumor immunotherapy has faced significant limitations over the past decades. Through analysis of existing literature, we hypothesize that these limitations may stem from obstacles in the recognition and binding process of TF-Ag by antibodies in cancer patients (Chen et al., 2024). To test this hypothesis, we conducted clinical sample testing and observed that serum levels of natural anti-TF-Ag antibodies (TF-Ab) were significantly lower in patients with papillary thyroid carcinoma (PTC) compared to healthy individuals, while sialic acid lectin levels showed no significant difference. This finding suggests that the functional activity of natural TF-Ab may remain largely intact.

Given that passive administration of TF-Ab has been proven to effectively inhibit the progression of TC, the therapeutic effect of optimizing TF-Ab is more likely to be attributed to TF-Ag exposure and recognition issues. Existing literature supports this notion, as the development of TF-Ag-targeted cancer vaccines depends on sialidase-mediated exposure of TF-Ag. To further elucidate the pathological role of TF-Ag in TC and facilitate the clinical application of TF-Ab-based therapies, we investigated the specific downstream pathological mechanism triggered by TF-Ag exposure to sialidase and found that Stratifin (SFN), a member of the 14-3-3 protein family, is a stable downstream interacting protein of TF-Ag.

SFN is a highly conserved soluble acidic protein that regulates cell cycle, cell growth and development, cell survival and death, gene transcription and other cell activities (Sharma et al., 2021). In recent years, SFN has been reported as a novel biomarker for a variety of cancers. Studies have found that SFN can promote the progression and development of early lung adenocarcinoma (Shiba-Ishii, 2021). For breast cancer, the biological characteristics of SFN have been confirmed to play an important role in the movement of breast cancer cells and depend on the pathological subtypes of breast cancer (Boudreau et al., 2013). In addition, it has been reported that overexpression of SFN in pancreatic, gastric and colorectal cancers is closely associated with poor prognosis (Huang et al., 2020). However, few studies have focused on the role of SFN in TC, and its potential molecular mechanisms and prognostic value in different subtypes of TC have not been explored and elucidated. This study aims to further explore the mechanism of SFN in TC and seek new directions for the diagnosis and treatment of advanced TC in the future.

## Materials and methods

### Human tissue samples

Human tissue samples were collected from patients with papillary thyroid carcinoma or locally advanced thyroid cancer from the First Affiliated Hospital of Kunming Medical University. Based on the findings from hematoxylin and eosin staining of sections for pathological diagnosis and histological types, three groups were included, papillary thyroid carcinoma (PTC), locally advanced thyroid cancer (Shonka et al., 2022), and healthy controls (histologically identified as normal thyroid tissue at a distance of more than 2 cm from the edge of the cancer). All tissue specimens were immediately frozen and transferred to the Kunming Institute of Biology, after which they were used to generate 10% buffer formalin-fixed and paraffin-embedded sections (N = 10), or protein extraction (N = 4). The paraffin sections were sliced into 5-μm-thick sections.

### Western Blotting

Use 500 μL of RIPA lysis buffer containing protease inhibitor (Servicebio) to lyse thyroid tissue; The protein concentration was determined using the BCA protein Assay Kit (Biyuntian); Then, the proteins extracted from the tissues were separated using 10% SDS-PAGE, and the SDS-PAGE gel was transferred onto the PVDF membrane (Millpore); The membrane was blocked with 5% BSA (solarbio), and the blocked PVDF membrane was transferred to the primary antibody β-actin (Proteintech, 1:25,000) for incubation. Subsequently, the secondary antibody HRP-labeled goat anti-rabbit IgG (invitrogen, 1:3000) and HRP-labeled goat anti-mouse IgG (invitrogen, 1:5000) were incubated, and color development was performed using the highly sensitive ECL luminescence kit (Affinity). The Voot biochemical luminescence imaging analysis system was exposed and images were collected. The gray values of the bands were obtained using the analysis software (ImageJ).

### Immunohistochemical analysis

Thyroid tissues were fixed overnight with 4% paraformaldehyde (phototreated), and after gradient dehydration with ethanol, they were embedded in paraffin and immunohistochemical staining was performed in 5 μm thick sections. The primary antibody was diluted according to the antibody instructions. The primary antibody used in the experiment was SFN(Proteintech, 1:1000). After incubating the primary antibody, enhanced enzyme-labeled sheep anti-mouse/rabbit IgG polymer was dropped in and incubated at 37 °C for 20 min. Rinse with PBS three times, add DAB (Seville) staining solution, rinse with PBS three times after obvious staining, stain in hematoxylin (Seville) for 5 min, and then dehydrate after differentiation and blue return. The slides were sealed with neutral gum (Seville), and the randomly selected areas were photographed and observed under an optical microscope.

### Cell culture

All TC cell lines (CAL-62, 8305C) were Cells were routinely cultured and maintained in Dulbecco’s Modified Eagle’s Medium (DMEM) with 10% Fetal Bovine Serum (FBS)(Gibco, 10099-141C, USA). And the cells were cultured in an incubator containing 5% CO_2_ at 37 °C. All cells were giving as presents by the Kunming Biology Institute, Chinese Academy (Kunming, China), cell authentication was confirmed by short tandem repeat profiling.

### Construction of SFN overexpression, knockdown and control stable transition strains

After overexpression of SFN, knockdown, construction and packaging of control lentiviral vectors in accordance with conventional methods, infection of OE-SFN lentivirus according to (OE-SFN: 8305C, 8305C OE-NC) Infection was carried out in groups (8305C infected with OE-NC lentivirus, cal62 sh-SFN:cal62 infected with sh-SFN lentivirus, cal62 sh-NC:cal62 infected with shNC lentivirus). 18–24 h before lentivirus infection of cells, adherend cells were spread in 6-well plates at a rate of 3 × 10^5^ per well. Make the number of cells at lentivirus transfection about 6 × 10^5^ per well; When the cell adhesion and fusion degree reaches 70%, replace the original medium with 2 mL of fresh medium containing 1 μg/ml polybrene and add 2 mL of virus suspension. Continue to culture at 37 °C for 8 h, and replace the medium containing the virus with fresh medium. Screening was conducted based on the selective markers (antibiotic resistance genes) of lentiviral vectors, and cell clones stably expressing SFN or controls were selected.

### *In vivo* and *in vitro* to study the effect of SFN expression on TC tumors

The cell proliferation was determined by the plate cloning experiment and the BeyoClick™ EdU-555 cell proliferation detection kit. The operation steps were all carried out in accordance with the operation steps in the conventional reagent manual. Staining was carried out in accordance with the standard methods of the Cell Cycle and Apoptosis Detection Kit (Biosharp) and the Annexin-V PE/7-AAD Apoptosis Detection Kit. 10^4^ cells of the stained samples were collected by BD FACSCalibur flow cytometer, and the results were obtained by Folwjo software. And the percentages of cells in each cycle were statistically analyzed. The comparison among the data was statistically analyzed using GraphPad Prism. Construct thyroid cancer cell lines with overexpression and silencing of SFN, and construct the BALB/c nude mouse transplanted tumor model. Regularly observe, take photos and record the changes in the volume of transplanted tumors. Mice were euthanized at the appropriate time points using cervical dislocation, in accordance with animal ethics guidelines, for sampling and analysis.

### Bioinformatics analysis

Following data mining and analysis of the ubiquitin-mediated proteolysis dataset using the GEPIA database, comprehensive bioinformatics analyses were performed. The processed data underwent further investigation through Venn diagram analysis and functional annotation, including Gene Ontology (GO) and KEGG pathway enrichment analyses. Subsequent analyses employed clusterProfiler for functional enrichment and STRING/Cytoscape for protein-protein interaction (PPI) network construction. Through systematic interpretation of these results, we explored the potential downstream regulatory mechanisms of SFN.

### GST pull-down assay

The GST-tagged target protein was expressed in E.coli BL21 (DE3) using pGEX vectors induced by 0.1–1 mM IPTG at 16 °C–37 °C for 4–6 h. Bacterial lysates were prepared in PBS containing 1 mM PMSF and protease inhibitors, followed by affinity purification using Glutathione Sepharose 4B beads. For binding assays, the purified GST-fusion protein was incubated with test proteins (cell lysates or purified proteins) in binding buffer (PBS containing 0.1%–1% NP-40/Triton X-100 and protease inhibitors) at 4 °C for 2 h or overnight. After extensive washing with cold wash buffer (PBS with 0.1% Triton X-100), bound proteins were eluted using 10–20 mM reduced glutathione (pH 8.0) or SDS loading buffer (95 °C, 5 min). Protein interactions were analyzed by SDS-PAGE and immunoblotting using specific antibodies. Control experiments included GST-only negative controls, known interaction partners as positive controls, and input samples to verify protein loading.

### Statistical analysis

Independent Student’s t-tests were used for statistical analyses between different groups. Experimental values are described as the mean ± standard deviation. P-values<0.05 indicated a significant difference between two groups.

## Results

### The downstream regulatory mechanisms of SFN primarily involve the TP53 signaling pathway

**FIGURE 1:**
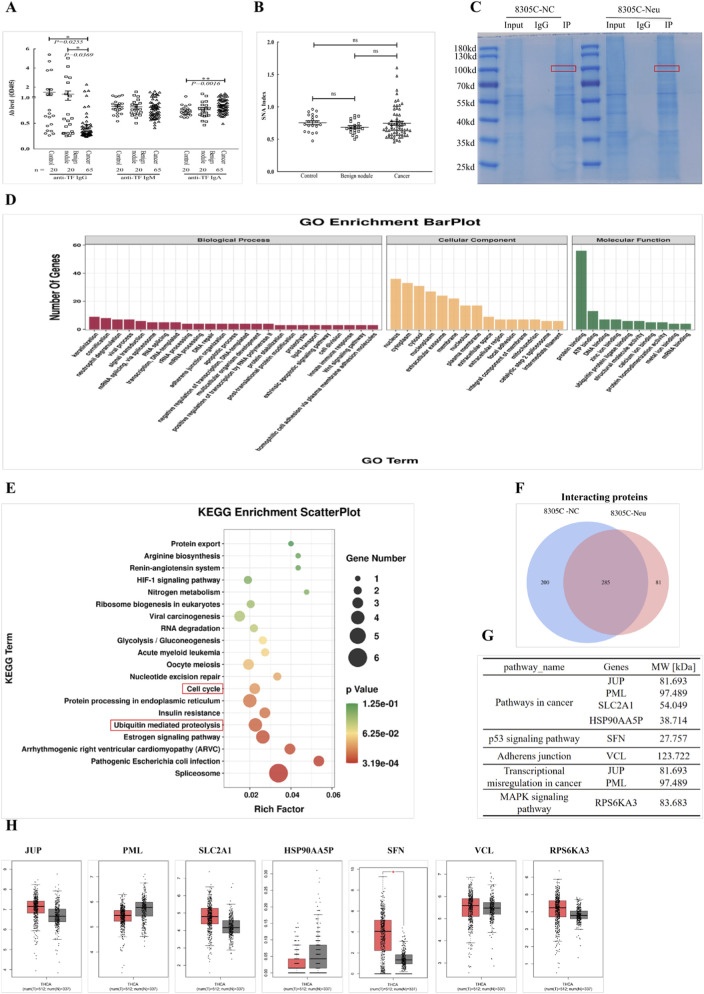
Analysis of the biological characteristics of SFN in TC. Note: **(A)** Expression levels of natural TF-Ab subtypes in different populations; **(B)** Detection of natural TF-Ab sialacidification levels in different populations (Control: N = 20, Benign nodule: N = 20, Cancer: N = 65); **(C)** IP-MS analyzed the molecular-interacting proteins that were stably and highly expressed after TF-Ag exposure. To understand the metabolic pathways, signaling pathways and other information of differentially expressed proteins after TF-Ag exposure, further bioinformatics analysis was adopted: **(D)** GO analysis of molecular-interacting proteins after TF-Ag exposure; **(E)** KEGG enrichment analysis of molecular-interacting proteins after TF-Ag exposure, where the dots represent the number of differentially expressed genes enriched in this pathway, and the larger the ratio, the greater the degree of enrichment; **(F)** Veen analysis was used to screen out the molecular-interacting proteins after TF-Ag exposure; **(G)** Signal pathway association diagram of molecular-interacting proteins with stable high expression after TF-Ag exposure after screening, SFN is mainly related to the TP53 signaling pathway; **(H)** Gepia database was used to mine and analyze the expression levels of the most significantly differentially expressed molecular interaction proteins in TC, among which only SFN was significantly highly expressed in TC.

ELISA results showed that the antigen recognition function of TF-Ab in PTC patients did not seem to have significant abnormalities, as there was no significant difference in the degree of sialacidification among them (Figures 1A,B). To gain deeper insights into the molecular mechanisms of TF-Ag in thyroid cancer (TC) metastasis and tumor progression, we conducted a systematic analysis of TF-Ag-interacting proteins using co-immunoprecipitation (Co-IP) combined with mass spectrometry (Figures 1C,D). GO functional clustering analysis (Figure 1E) demonstrated significant enrichment of TF-Ag-interacting proteins in several biological processes (BP), including stress fiber assembly, liver morphogenesis, integrin-mediated signaling pathway, regulation of fibroblast apoptosis, cytoplasmic mRNA processing body assembly, and trophoblast cell morphogenesis. At the cellular component (CC) level, these proteins were predominantly localized to membrane structures, cytosol, cytoplasm, extracellular exosomes, vesicles, and microtubules. Molecular function (MF) analysis revealed significant enrichment in protein binding, guanosine nucleotide exchange factor activity, microtubule binding, and calcium-dependent cysteine-type endopeptidase inhibitor activity domains. KEGG pathway enrichment analysis (Figure 1F) identified 20 significantly enriched pathways, with particularly strong relevance observed for cell cycle regulation and ubiquitin-mediated proteolysis pathways. Cross-referencing with the KEGG Pathway database (map05216) revealed several thyroid cancer-associated pathways, including p53 signaling pathway, pathways in cancer, adhere junction, MAPK signaling pathway, and transcriptional dysregulation in cancer. Validation of key pathway gene expression patterns using the GEPIA database (Figure 1G) showed that SFN exhibited significantly elevated expression in thyroid cancer (TC) tissues (*P* < 0.05), while JUP, PML, SLC2A1, HSP90AA5P, VCL and RPS6KA3 displayed no statistically significant differential expression (*P* > 0.05) (Figure 1H). Notably, existing research has established that dysregulation of the p53 signaling pathway plays a critical role in thyroid cancer progression. And our experimental results further indicate that SFN, as a stable and highly expressed interacting protein after TF-Ag exposure, it may play an important role in the occurrence and development of thyroid cancer by regulating the TP53 signaling pathway. This discovery provides novel insights into the molecular mechanisms of thyroid cancer metastasis and establishes a theoretical framework for developing targeted therapeutic strategies.

### SFN expression levels may be closely associated with the progression of thyroid cancer

**FIGURE 2:**
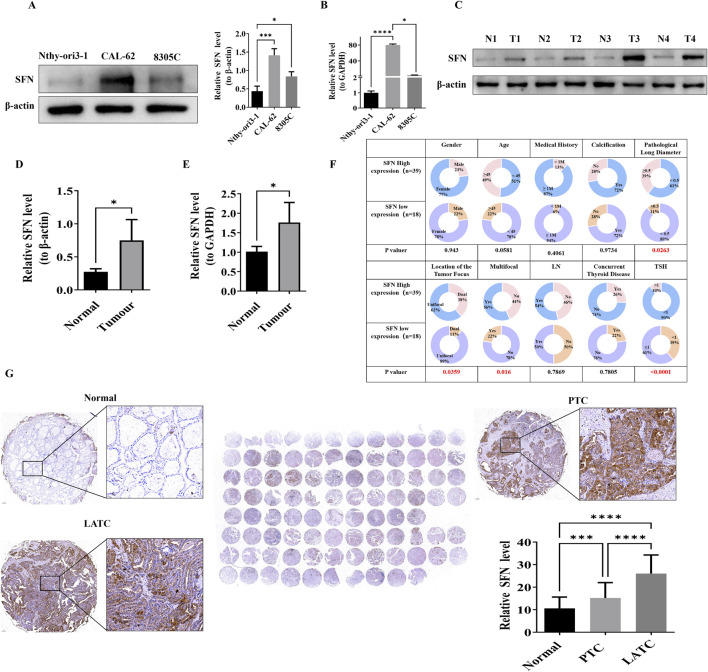
Detection of SFN expression level in TC. Note: **(A)** Western blotting was used to detect the expression differences of SFN in normal thyroid cells and thyroid cancer cell lines (8305c, CAL-62); **(B)** Statistical analysis chart of the results in Figure A, with the horizontal axis representing cell line grouping and the vertical axis representing the expression level of SFN, where ^*^ *P* < 0.05 and ^***^ *P* < 0.001; **(C)** QPCR was used to detect the expression differences of SFN in normal thyroid cells and thyroid cancer cell lines (8305c, CAL-62); **(D)** Western blotting was used to detect the difference in the expression level of SFN in adjacent thyroid tissues and thyroid cancer tissues (N = 4). **(E)** Statistical analysis chart of the results in Figure D, with the horizontal axis representing the tissue groups and the vertical axis representing the expression level of SFN, where ^*^ *P* < 0.05; **(F)** QPCR was used to detect the expression differences of SFN in adjacent thyroid tissues and thyroid cancer tissues (N = 8); **(G)** Correlation analysis between SFN expression levels and clinical characteristics of TC patients; **(H)** Immunohistochemistry was used to detect the difference in the expression level of SFN in adjacent thyroid tissues and thyroid cancer tissues (N: adjacent thyroid tissues = 34, thyroid cancer tissues = 58). In the statistical analysis graph, the horizontal axis represents the tissue groups and the vertical axis represents the expression level of SFN, where ^***^ *P* < 0.001.

Western blot analysis (Figures 2A–E) revealed significantly higher SFN expression in thyroid cancer cell lines (CAL-62 and 8305C) compared to the normal thyroid cell line Nthy-ori3-1 (*P* < 0.05). Immunohistochemical (IHC) staining (Figure 2F) further confirmed elevated SFN levels in thyroid carcinoma tissues relative to adjacent normal tissues (*P* < 0.05). Clinical correlation analysis of IHC data demonstrated statistically significant associations between high SFN expression (N = 29) and pathological features, including larger tumor size (long diameter), lesion location, multifocality, and elevated TSH levels (*P* < 0.05), whereas no correlations were observed with gender, age, or medical history (Figure 2G). Notably, SFN overexpression was not only a hallmark of thyroid cancer cells and tissues but was also more pronounced in locally advanced tumors compared to early-stage (T1) carcinomas (Supplementary Figure S1, N = 8), suggesting a potential link between SFN expression levels and clinical progression of the disease.

### Overexpression of SFN can significantly promote the development of TC

**FIGURE 3:**
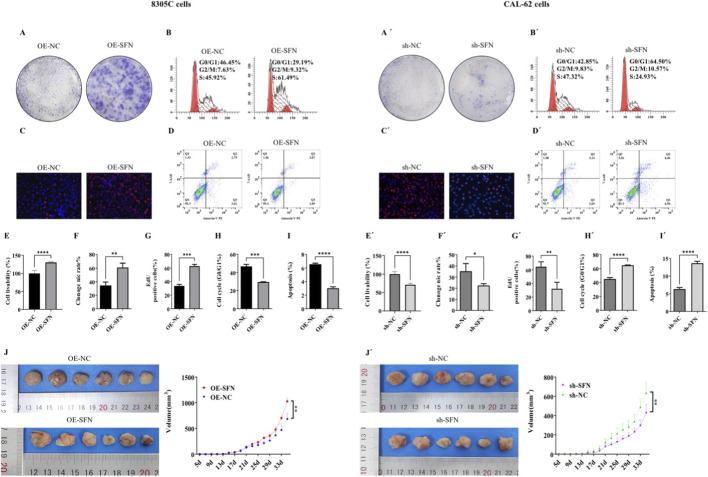
The effect of SFN expression regulation on the progression of TC detected by *in vitro* and *in vivo* experiments. Note: **(A/F)** the clone formation assay was used to detect the difference in cell proliferation of 8305C after SFN overexpression; **(A'/F')** the clone formation assay was used to detect the difference in cell proliferation of CAL-62 after knockdown of SFN. **(B/H)** flow cytometry was used to detect the cell cycle differences at 8305C after SFN overexpression; **(B'/H')** flow cytometry was used to detect whether there was a difference in the cell cycle of CAL-62 after knockdown of SFN. **(C/G)** tunel staining was used to detect the apoptotic differences of 8305C cells after SFN overexpression; **(C'/G')** tunel staining was used to detect the difference in apoptosis of CAL-62 cells after knockdown of SFN. **(D/I)** flow cytometry was used to detect the difference in apoptosis of 8305C cells after SFN overexpression. **(D'/I')** flow cytometry was used to detect the difference in apoptosis of CAL-62 cells after SFN knockdown. **(E/E')** cell proliferation assay was used to detect the difference in cell proliferation of 8305C after SFN overexpression, or of CAL-62 after SFN knockdown. **(J/J')** with the extension of transplantation time, the TC tumor volume in the SFN overexpression group was larger than that in the control group. The tumor volume in the SFN knockdown group was significantly smaller than that in the control group, ^**^ *P* < 0.01.

To investigate the mechanisms underlying SFN expression regulation in thyroid cancer (TC) progression, we established TC cell (8305C/CAL-62) models with SFN overexpression (OE-SFN) and knockdown (sh-SFN) using genetic manipulation techniques and conducted a series of cell biology functional experiments. In terms of proliferative capacity, the plate colony formation assay demonstrated that compared to the control group (8305C + OE-NC), the SFN overexpression group (8305C + OE-SFN) exhibited a significantly increased colony formation rate. Conversely, relative to the control group (Cal-62+sh-NC), the SFN knockdown group (Cal-62+sh-SFN) showed markedly reduced colony-forming ability (Figure 3A/F, 3A'/F′). The EdU proliferation assay further confirmed that SFN overexpression significantly enhanced cell proliferation, while SFN knockdown suppressed proliferative activity (Figure 3C/G, 3C'/G′). Cell cycle analysis revealed that SFN overexpression led to a decreased proportion of cells in G0/G1 phase and an increased proportion in S phase, indicating accelerated cell cycle progression. In contrast, SFN knockdown induced G0/G1 phase arrest and reduced S phase entry (Figure 3B/H, B'/H′). Flow cytometry demonstrated that SFN overexpression decreased the apoptosis rate in 8305C cells (*P* < 0.05), whereas SFN knockdown increased apoptosis in CAL-62 cells (*P* < 0.05) (Figure 3D/I, D'/I′). To validate these findings *in vivo*, we established a nude mouse xenograft model. The results showed that over time, tumor volume in the SFN overexpression group grew significantly faster than in the control group (*P* < 0.01), while tumor growth in the SFN knockdown group was markedly inhibited (*P* < 0.01) (Figure 3J/J′). These consistent results demonstrate that SFN functions as an oncogene in thyroid cancer. Its overexpression drives tumor progression by promoting cell proliferation, accelerating cell cycle progression, and inhibiting apoptosis, whereas targeted suppression of SFN expression effectively restrains malignant phenotypes. This discovery provides a novel potential therapeutic target for molecular-targeted intervention in locally advanced thyroid cancer.

### SFN affects TC tumor progression through regulation of the p53 signaling pathway

**FIGURE 4:**
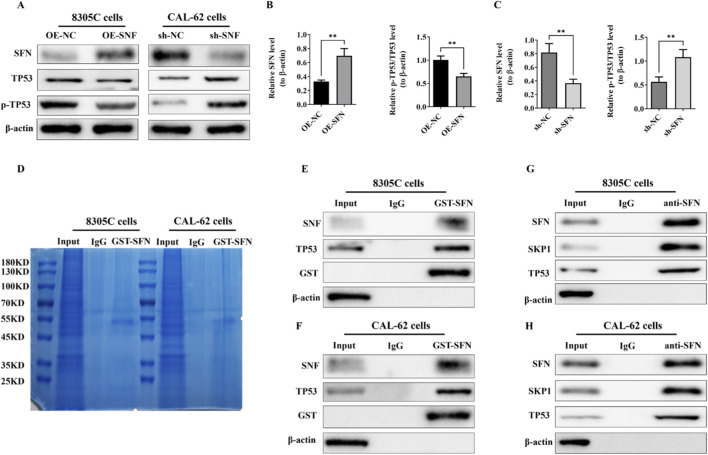
Verification of the regulatory relationship between SFN and TP53 expression. Note: **(A)** Western blotting was used to detect the expression differences of TP53 protein levels in normal thyroid cells with SFN overexpression/knockdown and thyroid cancer cell lines (8305C, CAL-62); **(B,C)** the result statistical analysis chart of Figure A, the horizontal axis represents the cell line grouping, and the vertical axis represents the detected protein expression level, where ^**^ *P* < 0.01, ^***^ *P* < 0.001, Figure B shows the 8305C cell line, and Figure C shows the CAL-62 cell line. **(D)** the GST pull-down experiment confirmed the targeted binding of SFN to TP53; **(E)** Western blotting was used to verify the targeted binding of SFN to TP53 in the 8305C cell line. **(F)** Western blotting was used to verify the targeted binding of SFN to TP53 in the CAL-62 cell line; **(G,H)** In 8305C and CAL-62 cells, the co-immunoprecipitation of Co (CO-IP) experiment was performed using SFN antibodies.

In our study, Western blotting results showed that in the 8305C cell line, SFN over expression (QE-SFN group) significantly downregulated p53 protein levels compared to the negative control group (QE-NC group) (*P* < 0.01). Conversely, in the CAL-62 cell line, SFN knock down (sh-SFN group) markedly upregulated p53 expression relative to the control group (sh-NC group) (*P* < 0.01)(Figure 4A/B/C). And based on preliminary bioinformatics analysis using the Gepia database suggesting that SFN(Stratifin) may primarily regulate the p53 signaling pathway, this study demonstrated the first experimental evidence in TC through the GST pull-down assay that there may be physical interactions between SFN and TP53 proteins as in other tumors (Figure 4D/E/F) (Huang et al., 2020), which was further confirmed under physiological conditions by Co-IP in both 8305C and CAL-62 cells, where SFN co-precipitated with TP53 and SKP1 (Figure 4G/H). Together, these results indicate that SFN negatively regulates p53 in thyroid cancer cells and may contribute to disease progression via the p53 signaling pathway, potentially in concert with SKP1-mediated mechanisms.

SFN may influence the progression of TC by regulating CSCs differentiation through SKP1-mediated ubiquitination and degradation of p53 expression.

**FIGURE 5:**
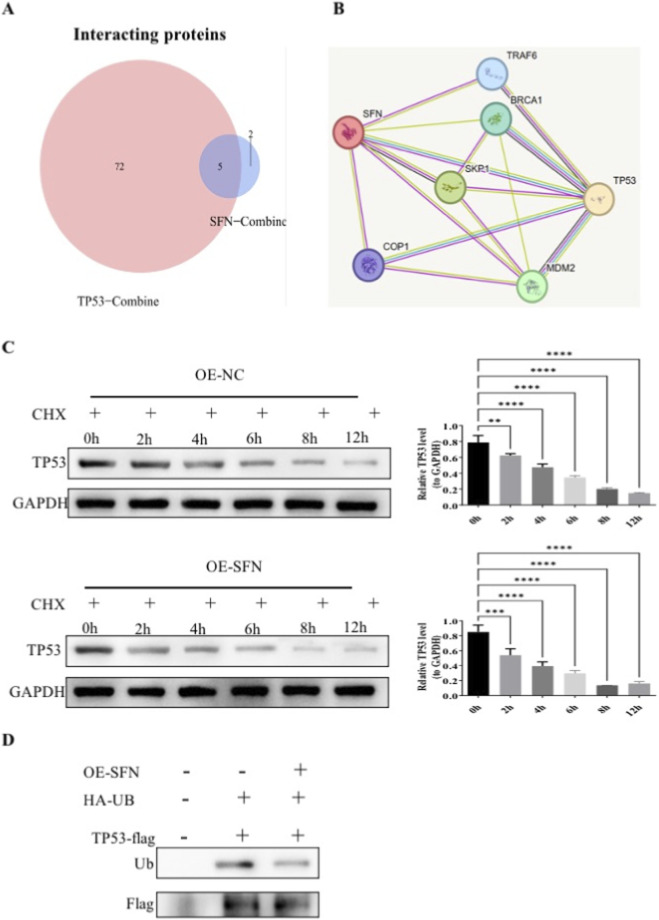
Exploration of the mechanism of TP53 ubiquitination modification mediated by SFN. Note: **(A)** Venn analysis on the ubiquitin-mediated protein degradation dataset to explore the ubiquitination regulatory proteins between SFN and TP53; **(B)** Association diagram of common ubiquitinated proteins of SFN and TP53; **(C)** Western blotting was used to detect overexpression of SFN, a CHX protein synthesis inhibitor was simultaneously added to detect the level of TP53 protein. **(D)** Verifying the ubiquitination level of TP53 protein by using ubiquitination labeling.

**FIGURE 6:**
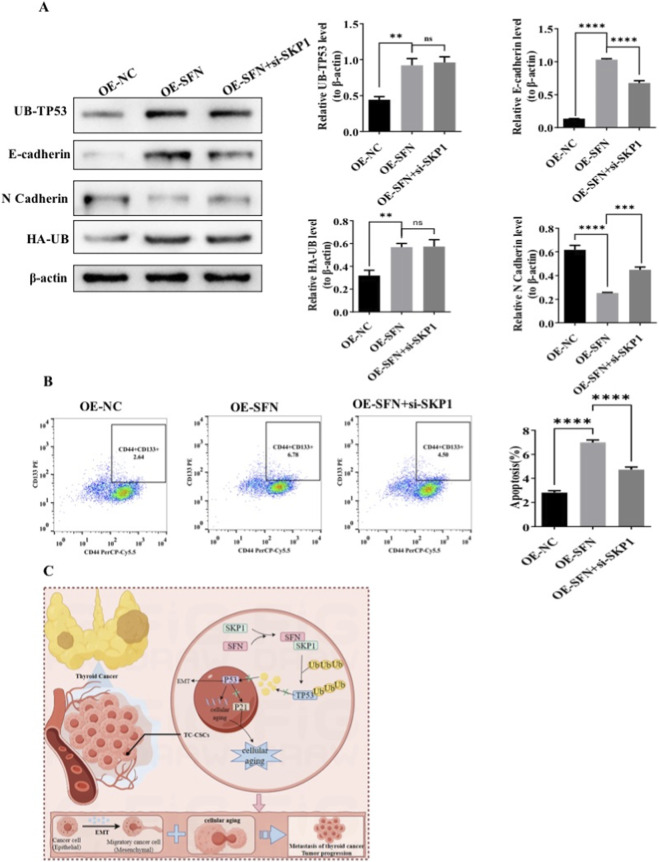
SFN may affects the differentiation ratio of TC-CSCs and the expression of EMT-related proteins in cells through SKP1-mediated TP53 ubiquitination modification. Note: **(A)** Western blotting was used to detect the ubiquitination level of TP53 and the expression of EMT-related proteins in cells after overexpression of SFN. Among them, overexpression of SFN could promote the increase of TP53 ubiquitination level and promote EMT, while interference with SKP1 could significantly reverse it. ^ns^ *P*>0.05, ^**^ *P* < 0.01, ^***^ *P* < 0.001, ^****^ *P* < 0.0001; **(B)** flow cytometry was used to detect changes in the differentiation ratio of TC-CSCs after overexpression of SFN. Among them, overexpression of SFN could promote an increase in the differentiation ratio of TC-CSCs, while interference with SKP1 could restore differentiation, ^****^ *P* < 0.0001. **(C)** The SKP1-SFN axis promotes epithelial-mesenchymal transition (EMT) of thyroid cancer stem cells (TC-CSCs) by regulating the P53/P21 pathway through ubiquitinating, ultimately accelerating the metastasis and tumor progression of thyroid cancer.

Based on bioinformatics analysis of the ubiquitin-mediated proteolytic system, we identified five key ubiquitination-regulating proteins (TRAF6, BRCA1, SKP1, COP1, and MDM2) shared between SFN and TP53, which may form a complex regulatory network (Figure 5). Using cycloheximide (CHX)-mediated translational inhibition combined with proteasome activity assays, Western blot analysis demonstrated that SFN overexpression significantly promoted ubiquitin-dependent degradation of TP53 protein and shortened its half-life. Further functional validation revealed that SFN, through this mechanism, markedly altered the differentiation dynamics of thyroid cancer stem cells (TC-CSCs) and induced distinct epithelial-mesenchymal transition (EMT) phenotypic changes. Notably, SKP1 overexpression effectively reversed SFN-mediated effects, not only restoring normal differentiation programs in CSCs but also downregulating EMT-related marker proteins (Figure 6). These findings uncover a novel mechanism by which SFN participates in TC-CSCs differentiation regulation through modulation of TP53 ubiquitination. This provides new molecular insights into the plasticity of thyroid cancer stem cells and the progression of EMT.

## Disscussion

CSCs are a subgroup of tumor cells that drive tumor initiation and cause tumor recurrence. The latest research proved that in most solid cancers, the presence of CSCs is closely related to tumor recurrence, spread and distant metastasis (Guha et al., 2023). CSCs generate tumors mainly through self-renewal and differentiation into multiple cell subtypes, and their activity is controlled by many intracellular and extracellular factors, which are considered to be key drug targets for the treatment of cancer and significantly improve the anti-tumor therapeutic effect. Previous studies have confirmed that ATC cells in locally advanced TC are derived from carcinogenic mutations in the thyroid stem cell genome (Xu et al., 2023), suggesting that locally advanced TC may be a malignant tumor driven by CSCs, and targeted CSCs therapy is expected to open up new therapeutic prospects for locally advanced TC.

In this study, our research identified SFN as a key molecular interaction partner with Thomsen-Friedenreich antigen (TF-Ag), a tumor-associated antigen strongly correlated with thyroid cancer (TC) metastasis, through immunoprecipitation (IP) coupled with mass spectrometry analysis. Notably, SFN exhibits significantly higher expression levels in locally advanced TC tissues compared to early-stage (T1) TC tissues. *In vitro* cellular experiments have demonstrated that modulation of SFN expression substantially impacts the biological behavior of TC cells and alters the differentiation ratio of cancer stem cells (CSCs). *In vivo* tumorigenicity assays using nude mouse models further confirmed that intervention targeting SFN expression effectively suppresses tumor progression. These findings collectively suggest that the SFN-regulated signaling axis may represent a key mechanistic pathway influencing the extensive metastasis observed in locally advanced TC, which may be achieved by regulating the differentiation of CSCs.

Existing evidence already supports that the expression of SFN can regulate the TP53-mediated cell cycle process (Falcicchio et al., 2020). In recent years, scholars such as Sharma have further confirmed that the SFN/TP53 signaling axis is crucial for the regulation of the tumor microenvironment (Sharma et al., 2021). In human cancer cell culture models, dysfunction of the TP53 pathway can lead to the escape of replicative senescence and extensive telomere erosion, ultimately resulting in dysregulation of CSCs differentiation and the development of immortal phenotypes to form tumors (Prime et al., 2023). Therefore, the dysregulation of CSCs differentiation in TC tumor cells may be closely related to the SFN/TP53 regulatory axis.

Emerging evidence suggests that SFN primarily modulates tumor metabolism by regulating gene expression through ubiquitination-mediated degradation of oncoproteins (Kim et al., 2018). The activation status of TP53 has been well-documented to correlate with its extended half-life, phosphorylation-induced conformational alterations, and various post-translational modifications (Paul et al., 2022; Xia et al., 2022). Our preliminary bioinformatics analysis revealed a regulatory network involving SFN, SKP1, and TP53. Notably, SKP1 serves as a crucial component of the SCF (SKP1-CUL1-F-box protein) ubiquitin ligase complex, which orchestrates the ubiquitination of proteins involved in cell cycle progression, signal transduction, and transcriptional regulation (Zeng et al., 2025). Previous studies have demonstrated that SCF E3 ligase expression correlates with tumor aggressiveness and unfavorable prognosis in thyroid cancer (Zeng et al., 2024). Consistently, co-immunoprecipitation using an SFN antibody demonstrated that SKP1 and TP53 co-precipitate with SFN, indicating that SFN forms a complex with SKP1 and TP53 under physiological conditions and suggesting that SKP1 may act as a key mediator in the SFN–TP53 ubiquitination axis.

Additionally, *in vitro* experiments demonstrated that SFN expression modulates TP53 levels and promotes TP53 protein ubiquitination. Investigations using CAL-62 cells (an ATC cell line) with SFN^+/+^ and SFN^−/−^ genotypes revealed that SFN influences the differentiation ratio of TC-CSCs and significantly alters EMT characteristics in CSCs. Remarkably, concurrent SKP1 knockdown in SFN-overexpressing cell lines restored CSC differentiation and reversed the EMT phenotype, suggesting that SFN may regulate TC-CSCs differentiation through SKP1-mediated ubiquitination of TP53.

A limitation of our study is that direct experimental evidence for the regulatory relationship between the Thomsen-Friedenreich antigen (TF-Ag) and Stratifin (SFN) has not yet been established. Based on supporting literature and our current findings, TF-Ag, as a tumor-associated glycoantigen, may potentially enhance SFN expression or stability indirectly through membrane receptor- or integrin-mediated signaling, activating PI3K–Akt–mTOR pathways, inhibiting GSK3β, and reducing SFN ubiquitination (Ma et al., 2021; Wang et al., 2022; Yang et al., 2024). TF-Ag may also influence tumor microenvironmental stress responses, activating TP53 and thereby increasing SFN transcription (Sharma et al., 2021). Our data and these observations suggest that TF-Ag is more likely to regulate SFN at the post-translational rather than transcriptional level, potentially through post-translational modifications such as glycosylation or by modulating protein–protein interactions. Nevertheless, the precise mechanisms remain to be experimentally validated, representing an important direction for future research.

Given our findings that SFN plays a critical role in regulating TP53 ubiquitination and CSC differentiation in thyroid cancer, targeting SFN represents a promising therapeutic strategy. Potential approaches include RNA interference to suppress SFN expression, small-molecule inhibitors to block its activity, or modulation of its ubiquitination pathway to destabilize SFN protein levels. Nonetheless, translating these strategies into clinical applications faces several challenges, including achieving target specificity, efficient intracellular delivery, and minimizing off-target effects. The development of safe and effective SFN-targeted therapies will therefore require rigorous preclinical validation and careful optimization to ensure clinical feasibility. Additionally, due to surgical complexity, the sample size for locally advanced TC in this study was limited (Supplementary Figure S1), and future studies should expand the cohort to further validate these results.

## Data Availability

The raw data supporting the conclusions of this article will be made available by the authors, without undue reservation.
