# Supplementary material for: Study on the mechanism of TP53 degradation by SFN ubiquitination affecting locally advanced TC progression
Source: Front Chem. 2026 Jun 29;14:1816666. doi: 10.3389/fchem.2026.1816666 (PMC13359834; doi:10.3389/fchem.2026.1816666)
Supplement: Supplementary file 1 [file DataSheet1.pdf]

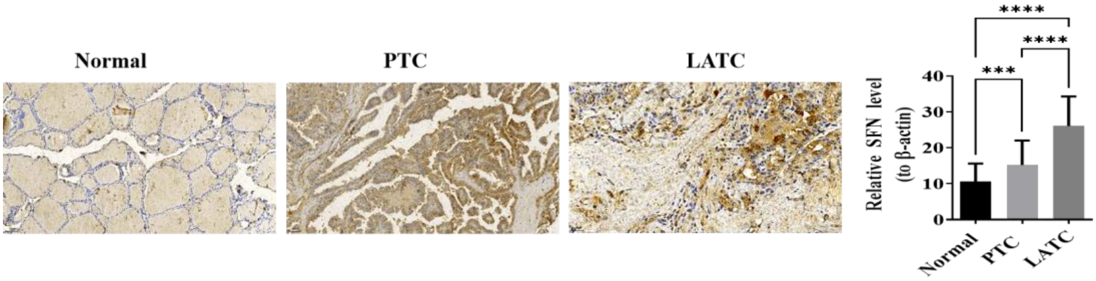

Supplemental Figure 1. Detection of SFN expression level in LATC.

Note: Immunohistochemistry was used to detect the difference in the expression level of SFN in adjacent thyroid tissues, PTC(T1 stage papillary thyroid cancer tissues) and LATC(locally advanced thyroid cancer) tissues(N=8). In the statistical analysis graph, the horizontal axis represents the tissue groups and the vertical axis represents the expression level of SFN, where $^{***}P<0.001$.
